# Visual affective classification by combining visual and text features

**DOI:** 10.1371/journal.pone.0183018

**Published:** 2017-08-29

**Authors:** Ningning Liu, Kai Wang, Xin Jin, Boyang Gao, Emmanuel Dellandréa, Liming Chen

**Affiliations:** 1 School of Information technology and Management, University of International Business and Economics, BJ, P.R.China; 2 School of Manufacturing Science and Engineering, Sichuan University, Chengdu, P.R.China; 3 Department of Computer Science and Technology, Beijing Electronic Science and Technology Institute, BJ, P.R.China; 4 Department of Advanced Robotics, Istituto Italiano di Tecnologia (IIT), via Morego, Genova, Italy; 5 LIRIS, Ecole Centrale de Lyon, Université de Lyon, CNRS, UMR5205, France; Boston Children’s Hospital / Harvard Medical School, UNITED STATES

## Abstract

Affective analysis of images in social networks has drawn much attention, and the texts surrounding images are proven to provide valuable semantic meanings about image content, which can hardly be represented by low-level visual features. In this paper, we propose a novel approach for visual affective classification (VAC) task. This approach combines visual representations along with novel text features through a fusion scheme based on Dempster-Shafer (D-S) Evidence Theory. Specifically, we not only investigate different types of visual features and fusion methods for VAC, but also propose textual features to effectively capture emotional semantics from the short text associated to images based on word similarity. Experiments are conducted on three public available databases: the International Affective Picture System (IAPS), the Artistic Photos and the MirFlickr Affect set. The results demonstrate that the proposed approach combining visual and textual features provides promising results for VAC task.

## Introduction

Visual object classification (VOC) targets on classification of objects in images at the cognitive level. By contrast, visual affective classification (VAC) aims at identifying the emotions that are expected to arise in image reviewers at the affective level, which proves to be extremely challenging due to the semantic gap between the low level visual features and the high level emotion-related concepts [1, 2]. VAC topic involves multiple research fields, including computer vision, pattern recognition, artificial intelligence, psychology, and cognitive science. Studies on psychology and affective computing [3–5] indicate that the prediction of emotions in image viewer is of subjectivity, which suggests that the emotions rely on individual feelings. The reason is that people from different backgrounds or cultures might perceive the same visual content quite differently. However, recent works on affective computing [3, 6–9] argue that certain features in images, as a universal validity to classify images in terms of affective concept, are believed to evoke some human feelings more easily, and have certain stability and generality across different people and different cultures.

In existing literatures, most works on VAC so far focus on investigating visual representations, e.g. color factors [10, 11], texture attributes [12], shape elements [9] as well as aesthetic features [13, 14]. Specifically, Colombo et al. [11] developed expressive and emotional level features based on Ittens theory [15] and semiotic principles. Machajdik et al. [5] investigated four groups of visual features for VAC including color, texture, composition and content. Liu et al. [16] proposed an emotion descriptors by using a novel affective probabilistic latent semantic analysis (affective-pLSA) model. Above works investigate visual representations for emotional concepts mainly by using traditional visual features [5], machine learning strategies [16] or human perceptual rules [11].

With the popular use of social networks in recent years, the increasing literatures have exposed rich resources of semantic information conveyed by online user generated content: the images and the associated texts(captions or tags) [9, 17–20]. Sivic et al. [18] investigated a text retrieval approach that can be successfully applied to VOC. Wang et al. [17] built a text-based feature by using the tags of an auxiliary dataset from internet, and demonstrated that it consistently improves performance on VOC problems. Liu et al. [21] proposed a multimodal approach effectively fusing visual and text modalities to predict various concepts (including 9 emotional ones) in images. All above methods improve the performance of different visual-based concept classification by making use of the texts from user generated content (UGC), as the texts surrounding an image (tags, discussions, group names) provide valuable information that can hardly be represented by the visual features [9]. In fact, the conventional visual features are hard to handle the unpredictability of objective concepts’ positions, sizes, appearances, lightings, and unusual camera angles, not to mention the emotional concepts. In a word, how to leverage the text and visual information to help perceiving the visual emotional semantics is one of the promising directions for VAC.

**Fig 1:**
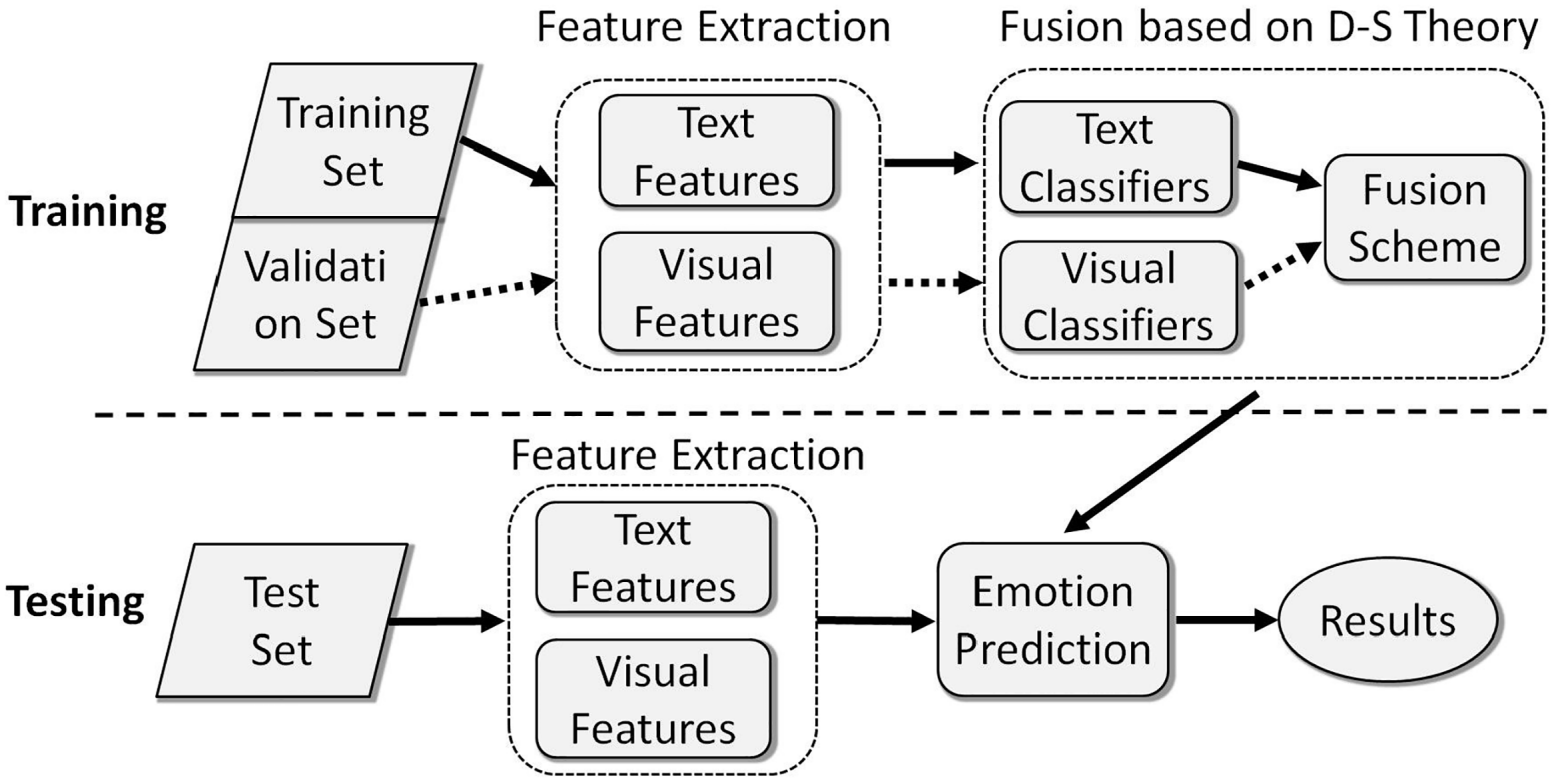
The framework of proposed approach. Note that if there is no tags associated to the image, only the visual classifiers are combined to predict the results.

In this paper, we target on proposing an effective approach combining visual and text information for VAC. Fig 1 shows the flowchart of our framework. For each of image, visual descriptors produce visual features for visual classifiers. Meanwhile, if available, the associated text is preprocessed to build the text features for text classifiers. All classifiers are then combined to predict the semantic emotion category of the input test image. In order to evaluate the effectiveness of proposed methods, we not only testify combining visual feature within two emotion models on the IAPS [22] and the Artistic Photos database [5], but also test fusing of visual and text features on the MirFlickr Affect set [8]. As a summary, the contributions of our paper can be summarized as following three aspects:

- We propose a late fusing scheme for VAC based on D-S Evidence Theory, whose interesting properties allow fusing different ambiguous sources of information. This scheme is proved to be efficient to fuse different features for VAC.
- We build a textual feature, namely the emotional Histogram of Textual Concepts (eHTC), to effectively capture emotional semantics for the short text, and we also try to measure the emotional metrics of text based on the Affective Norms for English Words(ANEW) data set.
- We investigate various visual features for VAC, including mid-level features related to aesthetic quality, harmony, dynamism *etc*., and evaluate their efficiency within two emotion models.

The rest of this paper is organized as follows. First, we describe the related works. Next, we present the proposed text features and other four groups of visual features for emotional semantics respectively. Then, we carry out the experiments and show the corresponding results. Finally, we give the discussion and draw the conclusion.

## Related works

**Fig 2:**
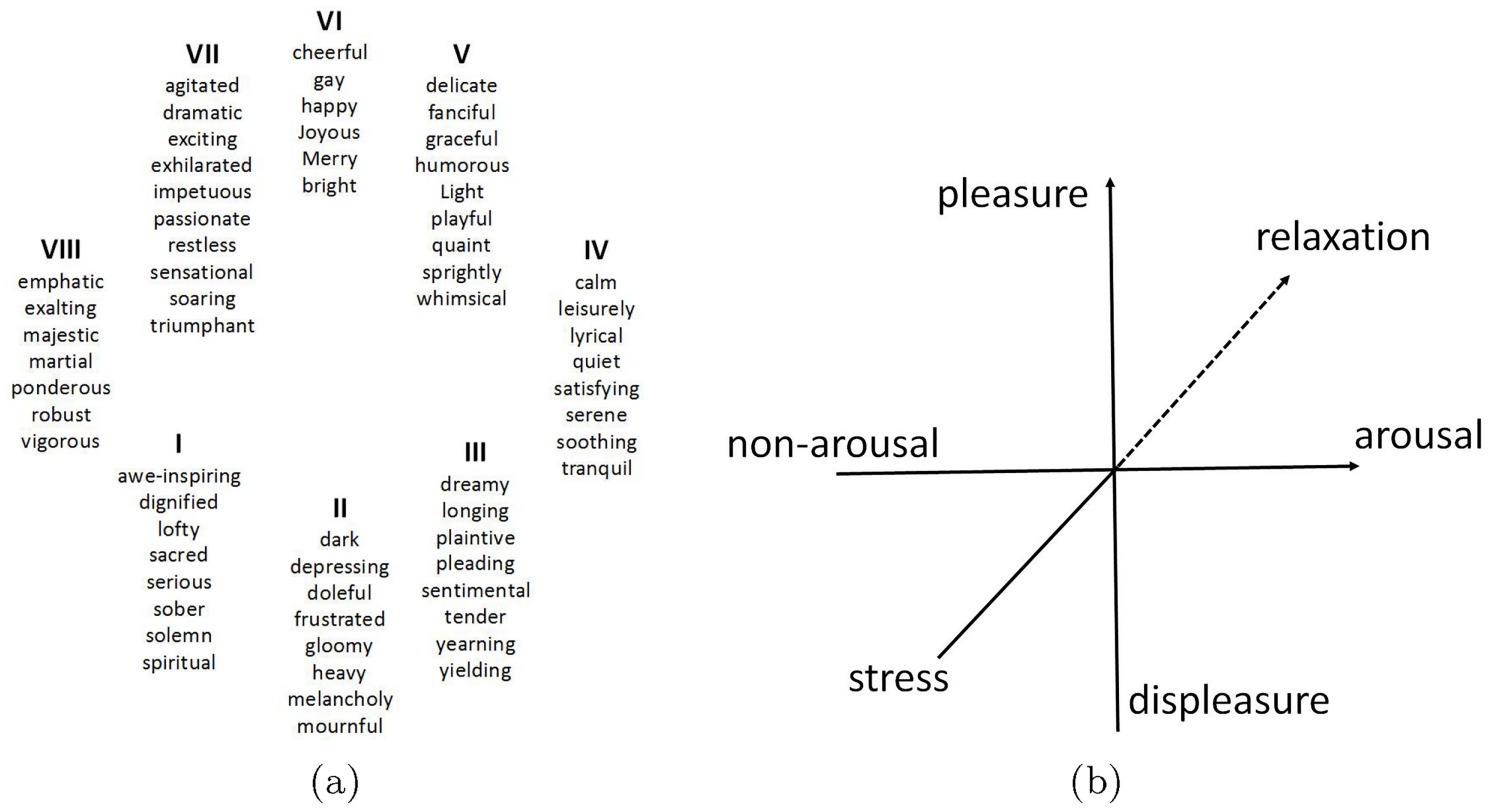
The two types of emotion models. While (a) is a discrete emotion model from Kate Hevners Adjective Circle [23], (b) is a dimensional model from Wundt’s [26].

As far as emotion recognition is concerned, researchers mainly focus on emotion recognition in audio (speech or music) and facial expressions (visual or 3D based). Limited contributions are concerned with the recognition of affective semantics carried by images, and a lot of issues need to be addressed particularly concerning following three fundamental problems: How to build emotion models to describe human moods in compute? How to extract features to represent high-level semantics and how to establish classification schemes to handle the distinctive characteristics of emotions [3]? Accordingly, the related works can be summarized as follows:

**The emotion models**. In literatures, several emotion models have been proposed and can generally be categorized into two types: the discrete one and the dimensional one. The discrete emotion models take adjectives or nouns to specify the emotions, such as happy, sadness, fear, anger, disgust and surprise. A common example is Kate Hevner’s Adjective Circle [23], as depicted in Fig 2(a). The dimensional models regard emotions as a coincidence of values on a number of different strategic dimensions [22], such as valence, arousal or dominance. A very early approach has been proposed by Wundt [23, 24], as shown in Fig 2(b). In practice, most works in VAC employ the discrete models, as it can be easily applied to image tagging or labeling [25], but they usually require a heavy dictionary, and they cannot represent a wide range of emotions compared with the dimensional ones, which allow percentage based ratings to assign specific emotion dimensions [3]. In this paper, both emotion models are employed to testify our approaches for VAC.

**The affective features**. The state-of-arts for VAC have so far proposed a large set of visual features, which generally can be divided into two types: the hierarchy approaches and the machine-learning approaches [27]. The approaches belonging to the first category build a hierarchical inference model based on the domain knowledge or rules. One of the initial works is from Colombo et al. [28], who proposed expressive and emotional level features based on Ittens theory [15] and semiotic principles. Ke et al. [14] proposed high level features for image aesthetic classification based on a group of principles, including simplicity, realism and basic photographic technique. On the other hand, the machine-learning based methods try to learn a mapping function between visual features and high level emotional semantics. Wang wei-ning et al. [29] firstly developed an orthogonal three-dimension emotional model using 12 pairs of emotional words, and then predict the emotional factor using SVM regression based on three fuzzy histograms. Machajdik et al. [5] employed two feature selection algorithms and investigated various features including color, texture, composition, and content features for affective image classification. In recent years, many works [30–36] based on deep learning [37] have been shown to achieve remarkable improvements on the performances of various VOC tasks [36, 38, 39], as these models can be trained to capture powerful features for visual objects. However, when it comes to emotions, the existing literatures based on deep learning mainly focus on face image based [40–42] or multiple physiological signals based emotion recognition [43]. The application of deep learning in VAC task is limited as it usually requires large amounts of data and time to train a robust model. Thus, the applicability of deep learning in domains are different but related to that of the training set [44]. In short, above approaches for VAC have largely demonstrated their effectiveness within different dataset, but the major shortcoming is still that their visual features are lack of descriptive power as regard to the high level emotional concepts.

As current visual representations for high-level visual concepts (e.g. objects, events, emotions) appear to be reaching the ceiling of performance, there exists an increasing works interested in web data mining [14, 17] or multimodal approaches [21, 45–48], which manage to utilize both the visual and associated text data from Internet. While the main approaches for representing the textual content are the word frequency statistic models (TF) with different variants (TF/IDF), these models have developed several extensions, including latent semantic analysis (LSA) [49], probabilistic LSA [50], and Latent Dirichlet allocation (LDA) [51]. As described in detail previously [21], the major drawback of these word frequency statistic-based approaches is lack of semantic sensitivity for three reasons: First, a text document is simply interpreted as an unordered collection of words, thereby disregarding grammar and even word order. Second, a text document is further summarized as a vector of term frequencies, thereby failing to capture the relatedness between words. Third, the tags for a given image from Internet is highly sparse (8.7 tags on average per image in MIR FLICKR), thereby hardly represented by the frequency statistic models. Recently, the word2vec [52] have provided state-of-the-art performances on many natural language processing (NLP) task [53, 54]. However, the different model parameters and different corpora sizes can greatly affect the quality of a word2vec model, which makes it hard to capture word sentiment from the small image tags data set [55]. As a consequence, popular text mining techniques (word2vec, LSTM) developed for text classification or retrieval are not applicable for the short text data directly. To tackle these problems, we propose novel textual representations, which can effectively handle the image tags and can improve the performance of VAC by effectively fusing with visual features.

**The classification scheme**. In the review of classification schemes for VAC, a number of works [16, 56, 57] build their classifying methods by employing the traditional classifiers. Yanulevskaya et al. [56] employed support vector machines (SVM) to build an emotion classification approach for art works. Guo et al. [57] proposed an emotion recognition system based on neuro-Hidden Markov Model (HMM) to classify the emotion contained in images. Liu et al. [16] built an emotion categorization system via a multilabel k-nearest-neighbor (KNN) classifier based on the visual descriptors. Among them, some works have tried to investigate fusion methods to improve the performance of VAC. One of works is from Machajdik [5], who simply concatenated low-level features to one vector and fed it to naive Bayers classifiers for VAC task. Ke [14] combined the quality metrics by linear fusion method for photo aesthetic classification. In recent years, many works [40, 42, 45] have proposed multimodal approaches to fuse visual and text data to analysis various concepts in images. Bänziger et al. [40] established the multimodal emotion recognition test (MERT) to measure the emotional competence in multimodal approaches that combining the visual and auditory sense modalities (audio/video, audio only, video only, still picture). M. Malinowski [45] proposed a multimodal approach for automatic question answering by combining semantic segmentations of real-world scenes with symbolic reasoning about questions in a Bayesian framework within Visual Turing Challenge [58, 59]. Ngiam et al. [42] proposed a novel application of deep networks to learn features by multiple modalities from multiple sources. Above works indicated that the performance of VAC or VOC task can be further improved by simple early fusing methods [5, 14] or by multimodal approaches [40, 42, 58, 59]. As emotions are high-level semantic concepts and by nature highly subjective and ambiguous, it is need to build a classification scheme to handle the information that may be uncertain, incomplete and leading to conflicts. In this paper, we manage to solve this issue by introducing a fusion method for VAC task based on the Evidence Theory, which allows to handle ambiguity and uncertainty in the emotion characteristics especially dealing with the small data set.

## Text features for emotional semantics

In this section, we first present a text feature for emotional semantics, namely emotional histogram of textual concepts(eHTC) which extends the histogram of textual concepts(HTC) [21] to capture the emotional tendency by employing an affective dictionary ANEW and a semantic similarity measurement. Moreover, we also propose a new text feature, namely emotional Metrics of Textual Concepts(eMTC) which measures the projection of tags in the three dimensional affective space [60] based on the affective ratings of the ANEW concepts.

### eHTC: emotional Histogram of Textual Concepts

**Fig 3:**
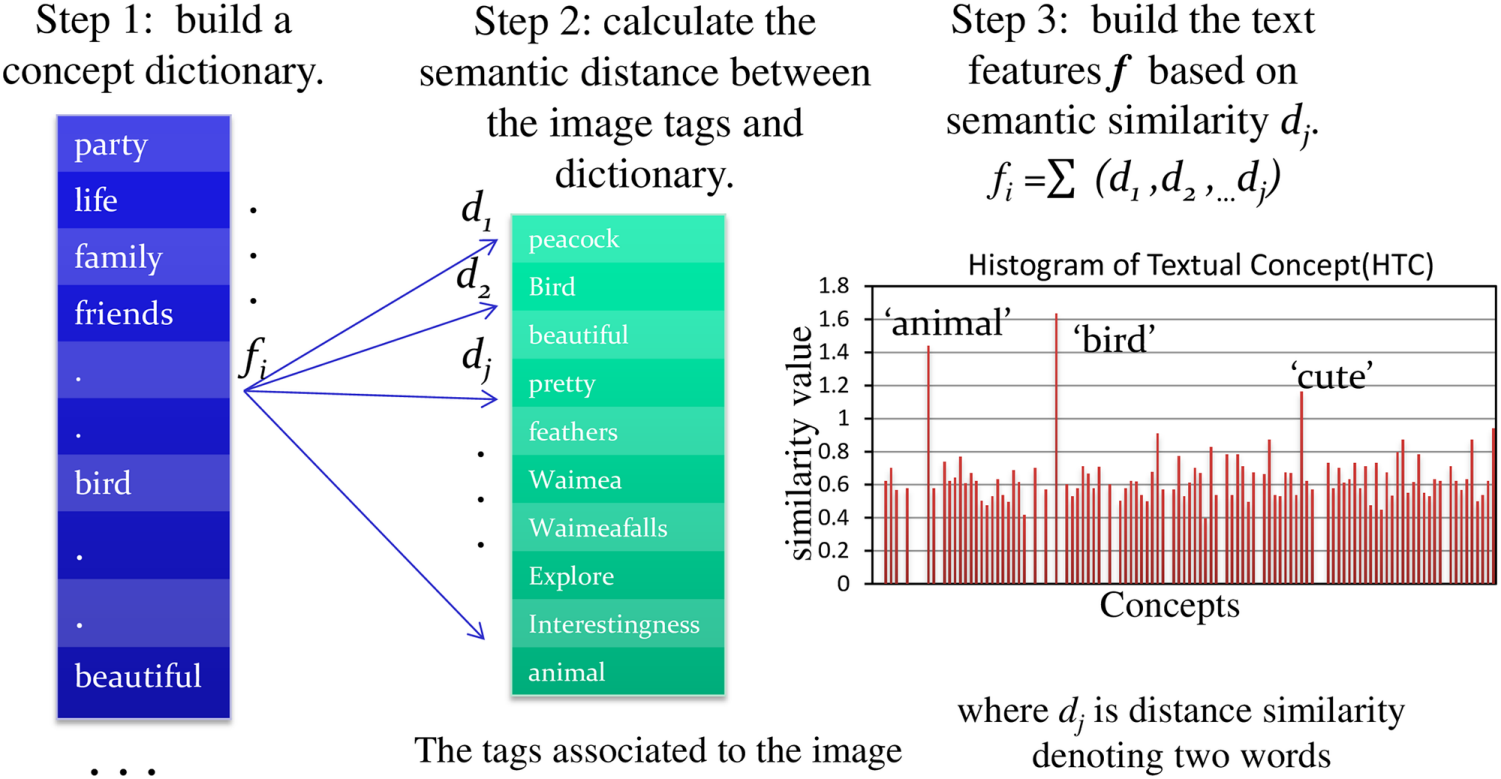
The three steps process of our HTC algorithm. As demonstrated in [21], the HTC describes the meaning of a word by its atoms, components, attributes, behavior, related ideas, *etc*., and is in clear contrast to the BoWs approaches where the relatedness of textual concepts are simply ignored as word terms are statistically counted. For instance, the concept of “peacock” can be described by “bird”, “beautiful”, “pretty”, “feathers”, “monsoon”, “animal”, “plumage”, *etc*.

In recent years, there is an strong increasement on sharing websites particularly related to photos and videos, and most of them (e.g. Flickr, Facebook, Weibo) allow users to share images and to contribute descriptions in the form of tags or captions. These texts provide valuable resources of information describing the visual data. Based on these data, Wang et al. [17] built a text-based feature (TF) by using an auxiliary dataset of images annotated with tags, and it improves the performance of VOC particularly when the training dataset is small. Mensink et al. [47] also employed the TF feature combing with the visual features to improve the performance of visual concept classification. In contrast to these conventional Bag-of-Words approaches, we have proposed HTC to capture the relatedness of semantic concepts through a three-step process as depicted in previously [21], showing in Fig 3.

In this paper, we propose the eHTC for VAC, which is to calculate a histogram of textual concepts towards an emotional dictionary, and each bin is the contribution of each word toward the underlying concept according to a predefined semantic similarity measurement. The calculation of eHTC needs a definition of dictionary and a proper words similarity. In practice, we use *D*_*ANEW*_ as the dictionary, which is being developed to provide a set of normative emotional ratings for a large number of words [61], and we employ the Resnik’s measurement as the words similarity, which uses the term probability based on the information content of a term distance. The algorithm of eHTC is detailed as following Algorithm 1:

**Algorithm 1**: **emotional Histogram of Textual Concepts** (**eHTC**)

**Input**: The tags *W* = {*w*_*t*_} and the dictionary *D*_*ANEW*_ = {*d*_*i*_} as *t* ∈ [1, T] and *i* ∈ [1, d].

**Output**: Histogram *f* composed of values *f*_*i*_ with 0 ≤ *f*_*i*_ ≤ 1 and *i* ∈ [1, d].

- Preprocess the tags by filtering with a stop-words list.
- If the image has no tags, return *f* as *f*_*i*_ = 0.5, ∀*i* ∈ [1, d].^1^
- For each word *w*_*t*_ in *W*:
  1. Compute the *dist*(*w*_*t*_, *d*_*i*_) between concepts *w*_*t*_ and *d*_*i*_, where *dist* is the Resnik measure.
  2. Obtain the matrix *M* as: *M*(*t*, *i*) = *dist*(*w*_*t*_, *d*_*i*_).
- Compute the feature *f* as: $f_{i}=\sum_{t=1}^{T}M\left(t,i\right)$, and normalize to [0 1] as: $f_{i}=f_{i}/\sum_{j=1}^{d}f_{j}$.

^1^ When an image has no tag, we set each bin value of eHTC as 0.5, which is at the middle between 0 (no connection to *d*_*i*_ in the dictionary) and 1 (sameness as *d*_*i*_ in the dictionary).

There are two main differences between HTC and eHTC. First, in contrast to HTC’s frequency-based words from the training data [21], the eHTC employs an emotional dictionary ANEW, which contains a relative large set of emotional words. Compared to other sentimental dictionaries e.g. POMS [62], SentiStrength [63], SentiWordNet [64], the ANEW is the most appropriate choice. Moreover, the eHTC uses the Resnik’s words similarity measurement [65], which performs well in a wide range of applications such as word sense disambiguation. In fact, we also evaluated other popular words similarity measurements [20] by using Natural language toolkit [66], such as Path [67], Wup [68] and Lin [69] distance measurements, but in our case the Resnik one proved to be the best choice.

Compared to the conventional term frequency-based features, the advantages of eHTC are multiple as the HTC’s [21]. First, for the sparse text such as image tags, eHTC offers a smooth description of the semantic measurements of user tags over a set of textual concepts defined by the dictionary. Second, for the case of polysemy or synonyms, eHTC helps disambiguate textual concepts according to the context. For example, the concept of “bank” can refer to a financial institution but also to the sloping land of a river. However, when a tag “bank” comes with a photo showing a financial institution with tags such as “finance”, “building”, “money”, *etc*., thereby clearly distinguishing the concept “bank” in finance from that of a river where correlated tags can be “water”, “boat”, “river”, *etc*. This is also the reason that we improve the performance of visual features and rank the first out of 80 runs within the ImageCLEF 2012 photo annotation challenge.

### eMTC: emotional Metrics of Textual Concepts

The eMTC is designed to measure the emotional metrics on valence, arousal, and dominance dimensions based on the ANEW set, in which each word is valuated with scores from 1 to 9 in terms of three affective dimensions *valence*(ranging from pleasant to unpleasant), *arousal*(ranging from calm to excited) and *dominance*(ranging from controlled to arbitrary). For instance, the “adorable” has a mean valence of 8.12, a mean arousal of 4.96 and a mean dominance of 6.00.

By using the affective ratings of the ANEW set, we compute the projection of an document on the three dimensional affective space, in terms of valence, arousal and dominance metrics by a linear combination between the ANEW concept’s ratings and the corresponding eHTC values. More precisely, based on the eHTC *f* extracted from a text, the emotional metrics of a text document in valence *m*_*v*_, arousal *m*_*a*_ and dominance *m*_*d*_ dimensions eMTC can be computed as follows in Algorithm 2:

**Algorithm 2**: **emotional Metrics of Textual Concepts** (**eMTC**)

**Input**: Tag data *W* = {*w*_*t*_} with *t* ∈ [1, T], dictionary *D*_*ANEW*_ = {*d*_*i*_} with *i* ∈ [1, d], the ratings of words in *D*_*ANEW*_ including valence *V*_*i*_, arousal *A*_*i*_ and control *C*_*i*_.

**Output**: Metrics in valence, arousal and dominance dimeansions (*m*_*v*_, *m*_*a*_, *m*_*c*_).

- Preprocess the text by using a stop-words filter.
- If the input image has no tags (*W* = ∅), return *m* with ∀*i* *m*_*i*_ = 0.5.
- Do for each word *w*_*t*_ ∈ *W*:
  1. Calculate *dist*(*w*_*t*_, *d*_*i*_), where *dist* is the Resnik measure between concepts *w*_*t*_ and *d*_*i*_.
  2. Obtain the semantic matrix *S* as: *S*(*t*, *i*) = *dist*(*w*_*t*_, *d*_*i*_).
- Calculate the eHTC feature *f* as: $f_{i}=\sum_{t=1}^{T}S\left(t,i\right)$, and normalize it to [0 1] as: $f_{i}=f_{i}/\sum_{j=1}^{d}f_{j}$.
- Calculate the eMTC feature *m* as: *m*_*v*_ = (1/*d*)∑_*i*_(*f*_*i*_.*V*_*i*_), *m*_*a*_ = (1/*d*)∑_*i*_(*f*_*i*_.*A*_*i*_) and *m*_*c*_ = (1/*d*)∑_*i*_(*f*_*i*_.*C*_*i*_).

## Visual features for emotional semantics

According to the study [5, 70], the VAC approaches are fundamentally different from the dominant VOC approaches, in which the SIFT-related features are the standard descriptors. The features based on global image statistics (global histograms) perform better than local image descriptors (bag-of-words models) for emotional categories [70]. In this paper, we compute a set of global features to represent the layout and the atmosphere of an image. All these features can be categorized into four groups: the color, texture, shape and mid-level, showing in Table 1:

**Table 1:** Summary of 4 group visual features.

Category	Features(Short name)	#	Short Description
*Color*	Color moments(color_Mom)	144	It is the three central moments(Mean, Standard deviation and Skewness) on HSV channels.
	Color histogram(color_Hist)	192	This histogram is concatenated from 64 bins on each HSV channel.
	Color correlograms(color_Corr)	256	It is a three-dimensional table expresses how the spatial correlation of color changes in a image.
Texture	Tamura(texture_Tamu)	3	Tamura [12] proposed visual features describing the coarseness, contrast, directionality.
	Grey level Co-occurrence matrix(texture_GCM)	16	It is defined as the distribution of co-occurring values at a given offset over an image.
	Local binary pattern(texture_LBP)	256	A compact multi-scale texture descriptor analysis of textures with multiple scales by combining neighborhoods with different sizes.
Shape	Histogram of line orientations(shape_Hist)	12	It is the number of lines in different orientations by using Hough transform [73].
Mid-level	Face information(mid_Face)	2	Number of faces in images and their emotion categories, based on Viola and Jones face detector [75] and Adaboost face expression recognition [76].
	Harmony(mid_Harm)	1	It is to describe the color harmony atmosphere of a image based on Itten’s color theory.
	Dynamism(mid_Dyna)	1	It is calculated as the ratio of oblique lines against horizontal and vertical ones.
	Y. Ke(mid_Ke)	5	We implement the aesthetic criterion from Ke [14] including: spatial distribution of edges, hue count, blur, contrast and brightness.
	R. Datta(mid_Datta)	44	We implement most of Datta’s aesthetic features [74] (44 of 56) except those related to the integrated region matching(IRM) calculation.

**Color**. According to [5], colors can be effectively used by artists to induce emotional effects. Studies [15] shows that HSV (Hue, Saturation, and Value) color space is more related to human color perceptions, and different color is associated with different emotions, such as red is connected to happiness, dynamism and power whereas its opposite color is green. In this paper, different features based on HSV color space are employed to describe color information in image including: color histograms, moments of color, and correlograms.

**Texture**. Textures in images also contain emotional expressions, e.g. Tamura features have been proven to correlate with human visual perception [5, 9]. In this paper, Tamura feature [12], co-occurrence [71], together with local binary pattern (LBP) [72] are employed to represent visual texture semantics.

**Shape**. Studies on artistic paintings have brought to the fore semantic meanings of shape and lines, and it is believed that shapes influence the degree of aesthetic beauty perception [11]. In this paper, the Hough transform is employed to build a histogram of line orientations in 12 different orientations [73].

**Mid-level**. We have proposed features to describe the harmony and dynamism characteristics in an image [8, 9]. The harmonic feature is computed according to Itten’s color theory [15], where colors are organized into a chromatic sphere and harmonious color positions on the sphere are connected thanks to regular polygons [21]. Meanwhile, a ratio has been proposed to characterize the dynamism in an image, defined as the numbers of oblique lines divided by the total number of lines in an image. Moreover, we implemented the works for image aesthetic classification [14, 74], which are expected to help measure the factor of pleasant in images, as a good aesthetic images usually can induce people pleasant feelings.

## Experiments and results

In this section, we conduct experiments on three datasets: the IAPS set (only images and the dataset is available at: http://csea.phhp.ufl.edu/media/requestform.html), the Artistic photo set (Only images and the dataset is available at: http://www.imageemotion.org/) and the MirFlickr Affect photos (Images and texts, and the dataset is available at: http://liris.cnrs.fr/membres?idn=edelland). After describing the experimental setup, we carry out several experiments with an in-depth analysis on: (1) the performance of visual features on IAPS and Artistic photo set; (2) the performance of visual and text features on MirFlickr Affect photos, and (3) the performance of combination approach based on D-S evidence theory.

### Affective image database

The available datasets for VAC are rather limited, and the available public database are described as below:

**IAPS**: According to [5, 22], the International Affective Picture System has being developed to provide a set of normative emotional stimuli for experimental investigations of emotion and attention. It is characterized along the dimensions of valence, arousal, and dominance. The image set contains various pictures depicting mutilations, snakes, insects, attack scenes, accidents, contamination, illness, loss, pollution, puppies, babies, and landscape scenes, among others. This data has been widely used in studies of emotion and VAC tasks [5, 8, 16, 27, 77].
**Artistic Photos**: According to [5], the artistic photos set was downloaded from an art sharing site [78], and was built to investigate whether the conscious use of colors and textures displayed by the artists can improve VAC. This dataset was obtained by using the emotion categories as search terms in the art sharing site, so the emotion category was determined by the artist who uploaded the photo. These photos were taken by people who attempt to evoke a certain emotion in the viewer of the photograph through the conscious manipulation of the image composition, lighting, colors, etc.
**MirFlickr Affect**: This affective dataset [79] was collected of about 2000 photographs selected from MIRFLICKR25000 Collection [80]. Compared to the IAPS and Abstract photo set, this dataset is much realistic, as the photos and tags are from the Flickr users uploaded and tagged. This collection supplies all original image tag data, which has an average number of 8.94 words per image. The emotion model of dataset relies on a dimensional view in two primary dimensions: valence one (ranging from pleasant to unpleasant) and arousal one(ranging from calm to excited), which improve the applicability for navigation and visualization [3]. In order to obtain the ground truth of affective space ratings in terms of valence and arousal, the selected 2000 images were rated from 1 to 9 by using a web-survey, where each one was assigned in average 20 times by 20 people. Meanwhile, we preprocess the ratings by abandoning instability samples, leaving with 1172 images [8].

### Experimental setup

The above databases are built for the studies focusing on emotional concepts, and are much professional compared with other benchmarks, e.g. PASCAL, ImageNET, ImageCLEF dataset. However, the main drawback is that these datasets are relatively small and highly unbalanced. Therefore, we need to carefully setup the experiments to get a convincing results.

- To evaluate the performance of visual features on IAPS and Artistic Photo set, we followed the work from Machajdik [5], who employed a discrete emotion model with 8 emotions: anger, awe, disgust, fear, sadness, excitement, contentment, and amusement [81]. Table 2 indicates that both datasets are relatively small (less than 400 images for each class) and with a quit unbalanced distributions. In order to leverage these problems, the experimental setup is done as follows: for the classifiers, we employ the Support Vector Machine framework (SVM) with Radial Basis Functions (RBFs) using one against all scheme, and choose the average true positive rate (ATPR) per class over the positive and negative classes as the evaluation measurement defined by [5]. We carry out the experiments in 5-fold cross validation, and evaluate the validity of visual features on IAPS and Artistic Photo sets.
- To evaluate the performance of visual and text features on MirFlickr Affect dataset, we firstly build six classes by equally dividing each dimension into three levels: low, neutral and high, showing in Table 3. The experiments are then set as follows: we build six SVM classifiers for each classes using one against all scheme. More specifically, LIBSVM tool [82] are employed, and the input features are normalized to train the RBF kernel based SVM classifiers, which produce measurement vector as the degree of input belongs to different classes. To obtain reliable results, we measure the ATPR performance of visual and text features within 5-fold cross validation.
- To combine the features, we employ a powerful classifier combination technique based on the D-S evidence theory, whose interesting properties allow to fuse different ambiguous sources of information. The experimental setup is carried out as follows: First, we extract features on MirFlickr Affect set and train the SVM classifiers on a training set (50% data set), which produces measurement vector as the degree of input belongs to different classes. Then, we define the belief function *d*_*n*_(*θ*_*k*_) according to the following formulas:
  $$\begin{array}{c} m_{n}\left(\theta_{k}\right)=d_{n}\left(\theta_{k}\right)/\left(\sum_{k}d_{n}\left(\theta_{k}\right)+0.5\right) \end{array}$$ (1)
  where $d_{n}\left(\theta_{k}\right)=\exp\left(-{∥w_{k}^{n}-y^{n}∥}^{2}\right)$, $w_{k}^{n}$ is a reference vector and will be initialized randomly. Finally, as inspired by the Al-Ani’s work [83], the classifiers are combined by adjusting $w_{k}^{n}$ so that minimizing the mean square error (MSE) of the combined classification results *z*_*k*_ as
  $$\begin{array}{c} Err=||z-{t||}^{2} \end{array}$$ (2)
  $$\begin{array}{c} z_{k}=⊕m_{n}\left(\theta_{k}\right) \end{array}$$ (3)
  where *t* is the target output. Meanwhile, we make a comparison with different types of combination approaches, including:
  $$\begin{array}{c} mean\;-\;score:z_{k}=\frac{1}{N}\sum_{n=1}^{N}y_{k}^{n} \end{array}$$ (4)
  $$\begin{array}{c} majority\;-\;voting:z_{k}=argmax\left(y_{k}^{1},y_{k}^{2},...,y_{k}^{N}\right) \end{array}$$ (5)
  where $y_{k}^{n}$ represent the *k*^*th*^ measurement of classifier *c*_*n*_. In order to testify the fusion methods for VAC, we combine the visual features for IAPS and Artistic Photo set, and fuse the visual and text features for MirFlickr Affect dataset.

**Table 2:** Number of images per dataset and emotion category in IAPS and artistic photo.

	*Amusement*	*Anger*	*Awe*	*Contentment*	*Disgust*	*Excitement*	*Fear*	*Sad*	*sum*
IAPS*	37	8	54	63	74	55	42	61	394
Artistic P.	101	77	103	70	70	105	115	166	807

Please note that IAPS* is a subset of 394 images from the IAPS dataset.

**Table 3:** The description of the MirFlickr affect dataset.

Database	Size	Text	LV	NV	HV	LA	NA	HA
MirFlickr Affect dataset	1172	8.93/image	257	413	502	261	693	218

Please note that LA is the acronym of low arousal, NA is neutral arousal, HA is high arousal, LV is low valence, NV is neutral valence and HV is high valence.

### Performance of visual features on IAPS and artistic photos

**Fig 4:**
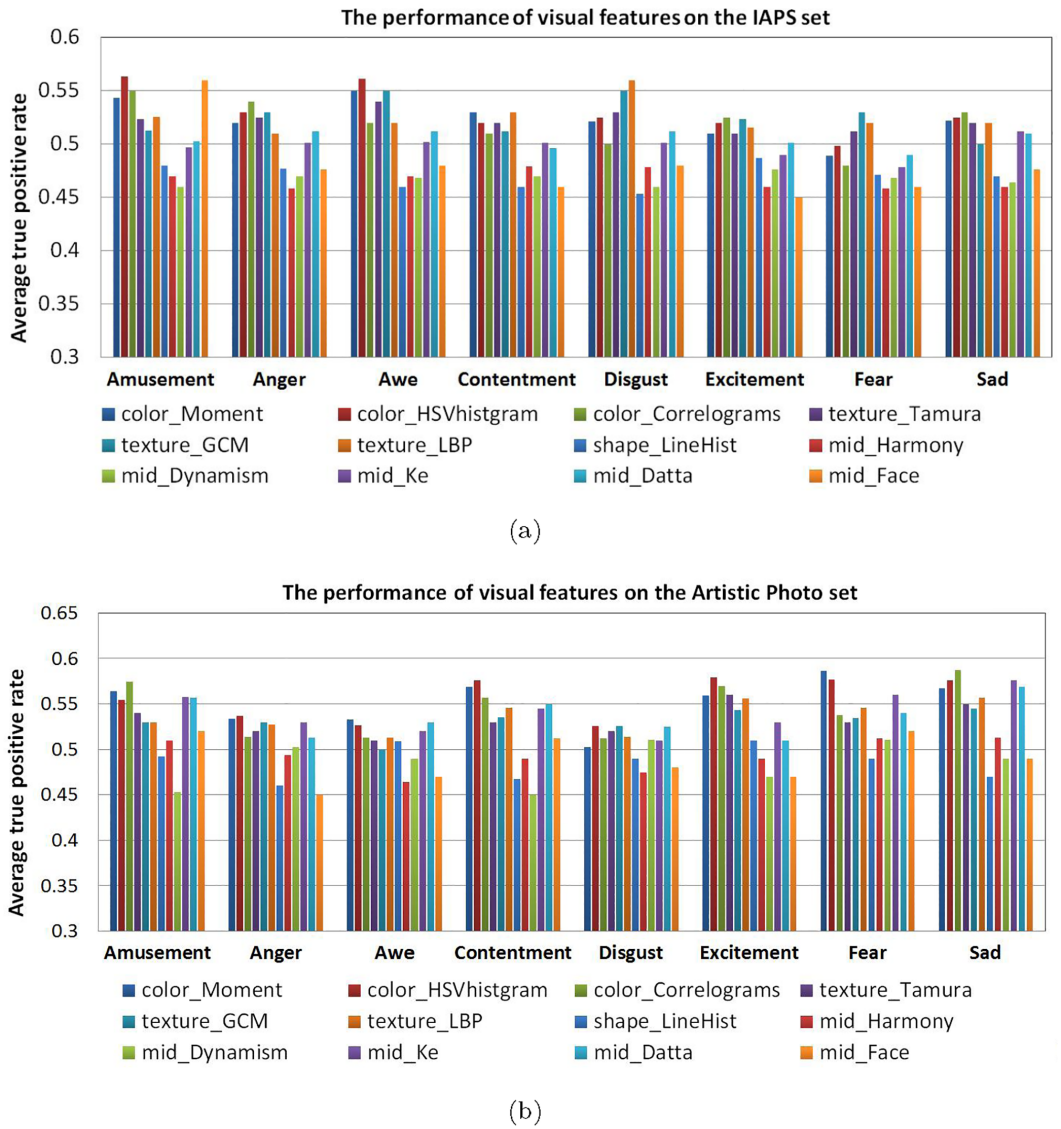
Performance of each visual feature on IAPS (a) & artistic photo (b).

Fig 4 shows the performance of each visual feature on the IAPS (a) and Artistic Photos sets (b) respectively. Fig 4(a) shows the results of the IAPS, and we can see that the texture and color features with average 55% ATPR perform better than the shape and mid-level ones for most categories. The reason is that the shape and mid-level descriptors are low dimensions and with low discriminative power to classify the high-level concepts. Also, we can see that the mid-level feature face information (*mid*_*Face*) with 56% ATPR is among one of the most powerful features for the “Amusement” category. The texture-related features (*texture*_*GCM*, *texture*_*LBP*) perform better than the others especially for “Disgust” and “Fear” categories in the IAPS set. The reason is that the images in the IAPS set are highly content related, for example, the “Amusement” images usually include happy people with smiling faces which can easily be identified by face detector, whereas “Fear” and “Disgust” images often show insects, snakes or injuries with certain textures which can be distinguished by texture descriptors. However, the face information(*mid*_*Face*) does not make a contribution to other classes in which few images have human face. Fig 4(b) shows the performance of each visual features on the Artistic Photos. We can see that the color features(*color*_*Moment*, *color*_*HSVhistogram*) are the most effective ones among the visual features, following by aesthetic-related features(*mid*_*Ke*, *mid*_*Datta*) and texture features(*texture*_*LBP*). This indicates that the color information plays important role for classification of the artistic photos. Meanwhile, the face information (*mid*_*Face*) fails to detect “Amusement” and other categories in the Artistic Photos set, as there is no strong correlation between faces and classes in the set.

### Performance of visual and text features on MirFlickr affect set

**Fig 5:**
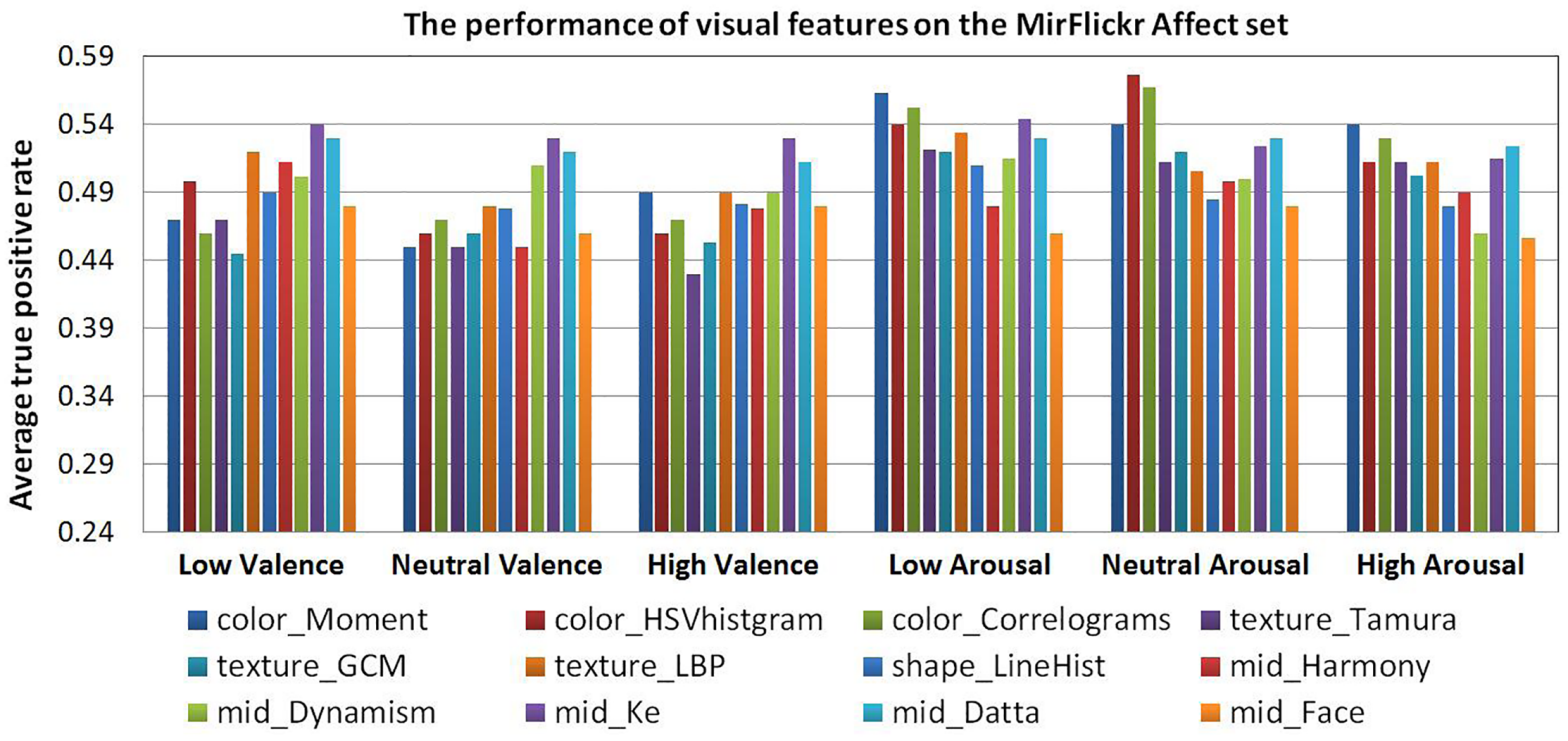
Performance of each visual features on MirFlickr affect sets.

Fig 5 shows the performance of visual features on the MirFlickr Affect set. We can see that the mid-level aesthetic features (*mid*_*Datta*, *mid*_*Ke*) with average 50% APTR perform better on valence dimension, while the color features (*color*_*HSVhistogram*, *color*_*Correlograms*) with average 56% APTR perform better on arousal dimension. It can be interpreted as the aesthetics more likely influence human pleasant feelings that related to the valence, and the colors more probably induce human active emotions that related to arousal. This is also confirmed by Machajdik’s work [5].

**Fig 6:**
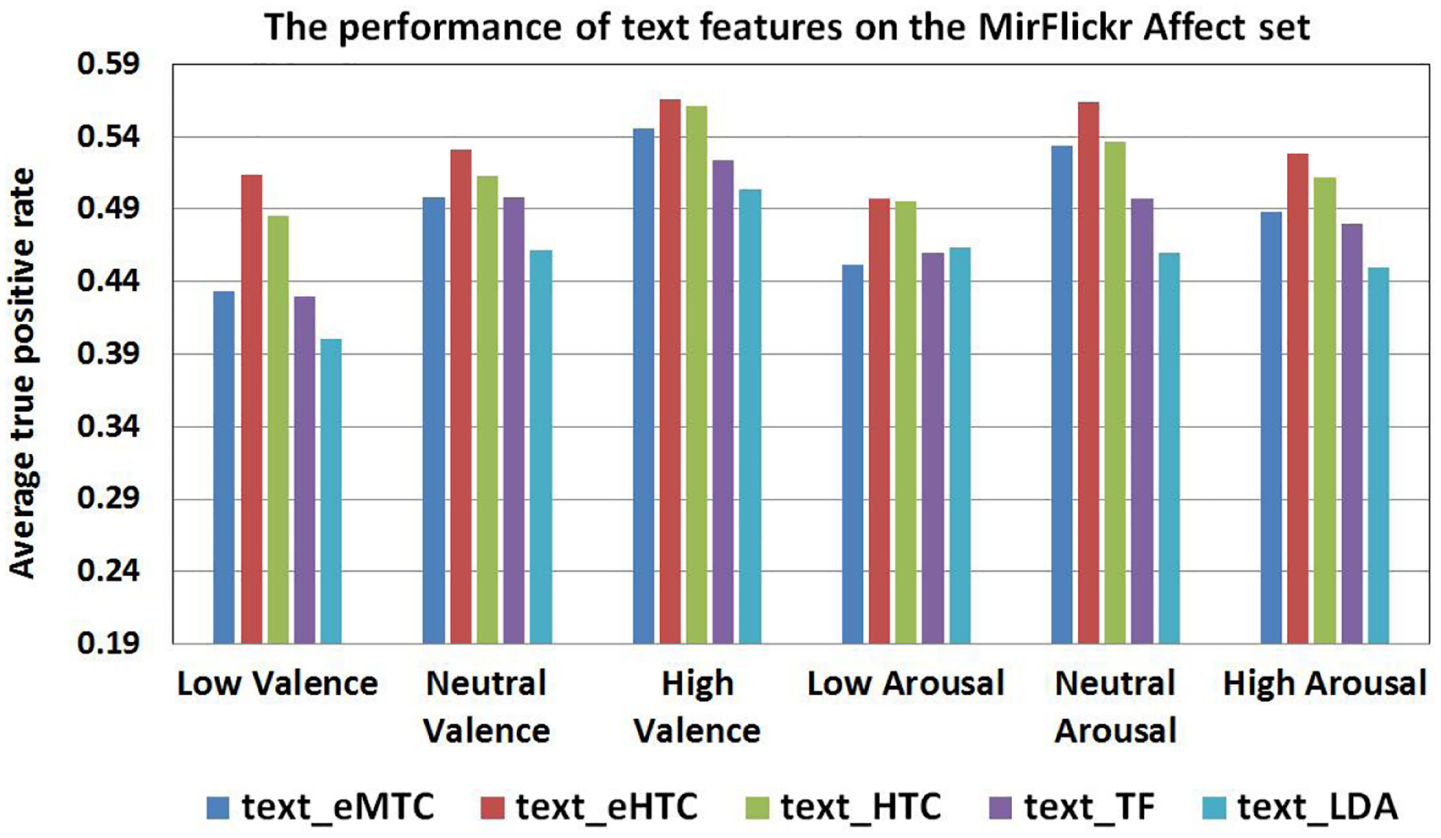
Performance of text features on MirFlickr affect set.

Fig 6 shows the performance of text features on MirFlickr Affect set. We can see that the eHTC achieves the best performance with average 57% ATPR, which is better than HTC with average 54% ATPR. The reason is that firstly the dictionary of eHTC is much more interrelated to the affective concepts compared to the HTC’s word frequency based dictionary, and secondly the Resnik’s words similarity measurement also contributes to strengthen the discriminative power on VAC concepts. It also shows that the performance of eMTC is among the lowest one in terms of ATPR and is unfortunately closing to random. It can be explained as the empiric ratings of the ANEW words are highly subjective, and many terms have high standard deviations, which imply less confidence associated to the rating values. Meanwhile, we make a comparison with other popular text features, such as the TF and the Latent Dirichlet allocation (LDA) topic model [51]. However, the results on Fig 6 indicate that the LDA, learned with 64 topics, do not work well with the image tags and receives the worst performance with average 46% ATPR. The main reason lies in the fact that image tags or captions are generally short with less than 10 tags per image (e.g 7.8 words for MirFlickr tags). This makes TF feature very sparse with many zeros, and causes inadequate training on LDA topic model. In a word, the results confirm that HTC [21] and its variant eHTC are proven to be effective in particular when handling short texts in social networks.

### Performance of combining features based on D-S evidence theory

In this section, we firstly show the performance of combining visual features on IAPS and Artistic Photo set, then present the performance of fusing visual and text features on MirFlickr Affect dataset. Meanwhile, we make a comparison with the state-of-arts.

**Fig 7:**
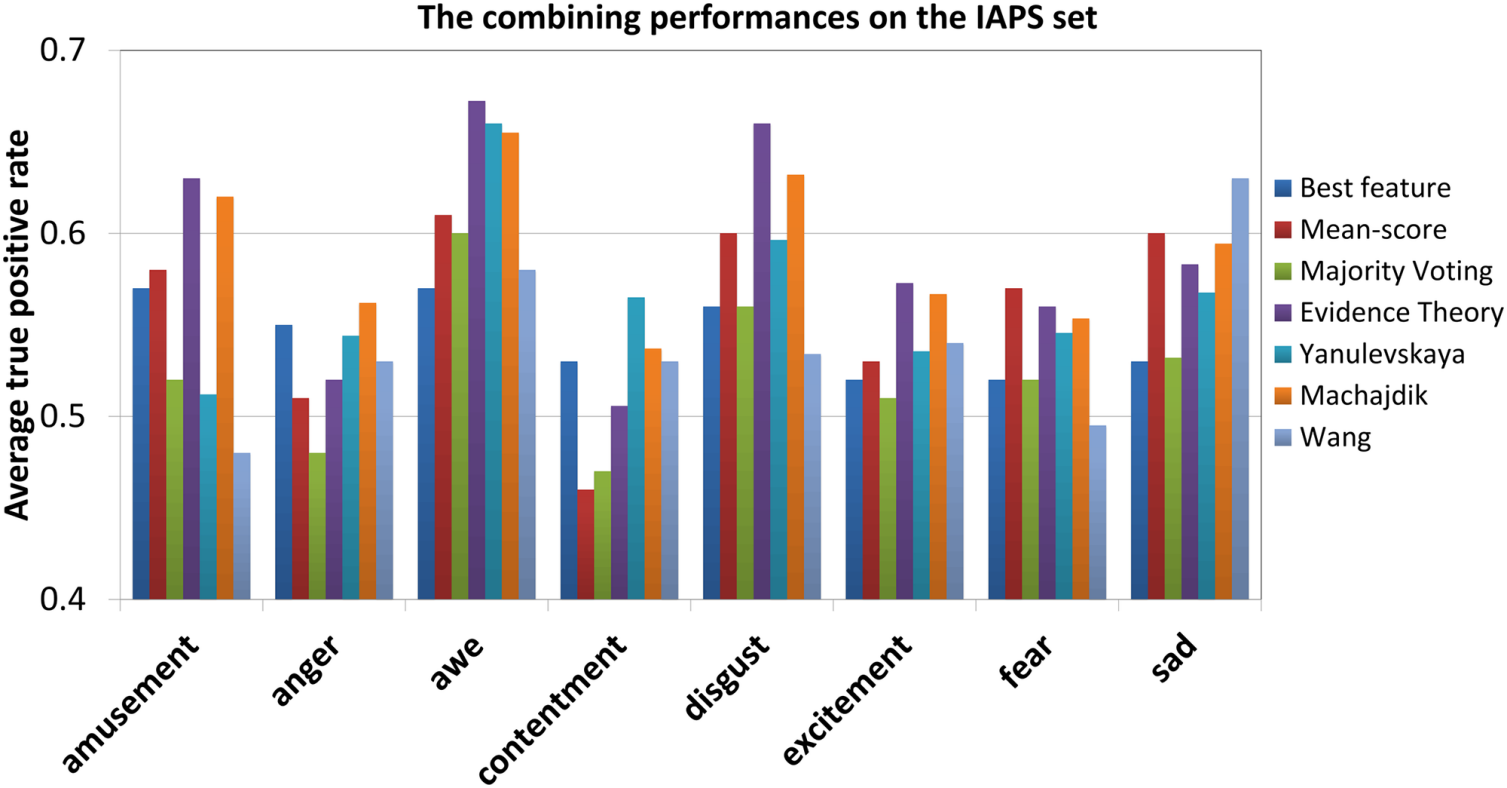
Performance of the various combination methods on the IAPS dataset.

Fig 7 shows the results of different fusion methods on IAPS set and the comparison with the state-of-arts [5, 29, 77]. We can see that the performance show an improvement by employing fusion methods compared to the case of best feature used, especially highlighted for “amusement”, “awe”, “disgust” and “sad” concepts with average 5% ATPR improvement. More specifically, the D-S evidence theory method obtains the best result with average 61% ATPR compared with other conventional methods, e.g. mean-score and majority voting. Also, it outperforms the state-of-arts by Yanulevskaya [77] and Machajdik [5] for five of eight categories (except “anger”, “contentment” and “sad”). All these indicate that the D-S evidence theory fusion approach is suitable for VAC task, as it is based on adjusting the evidence of different classifiers by minimizing the MSE of training data. The accurate estimation of evidence of each classifier will lead to minimizing the MSE of the combined results, and hence resolve the conflicts between classifiers. However, one should be noted that for “anger” category, the result is only a bit better than random chance(52% for one-versus-all), which can be expected in this challenging task with such a small set of training images.

**Fig 8:**
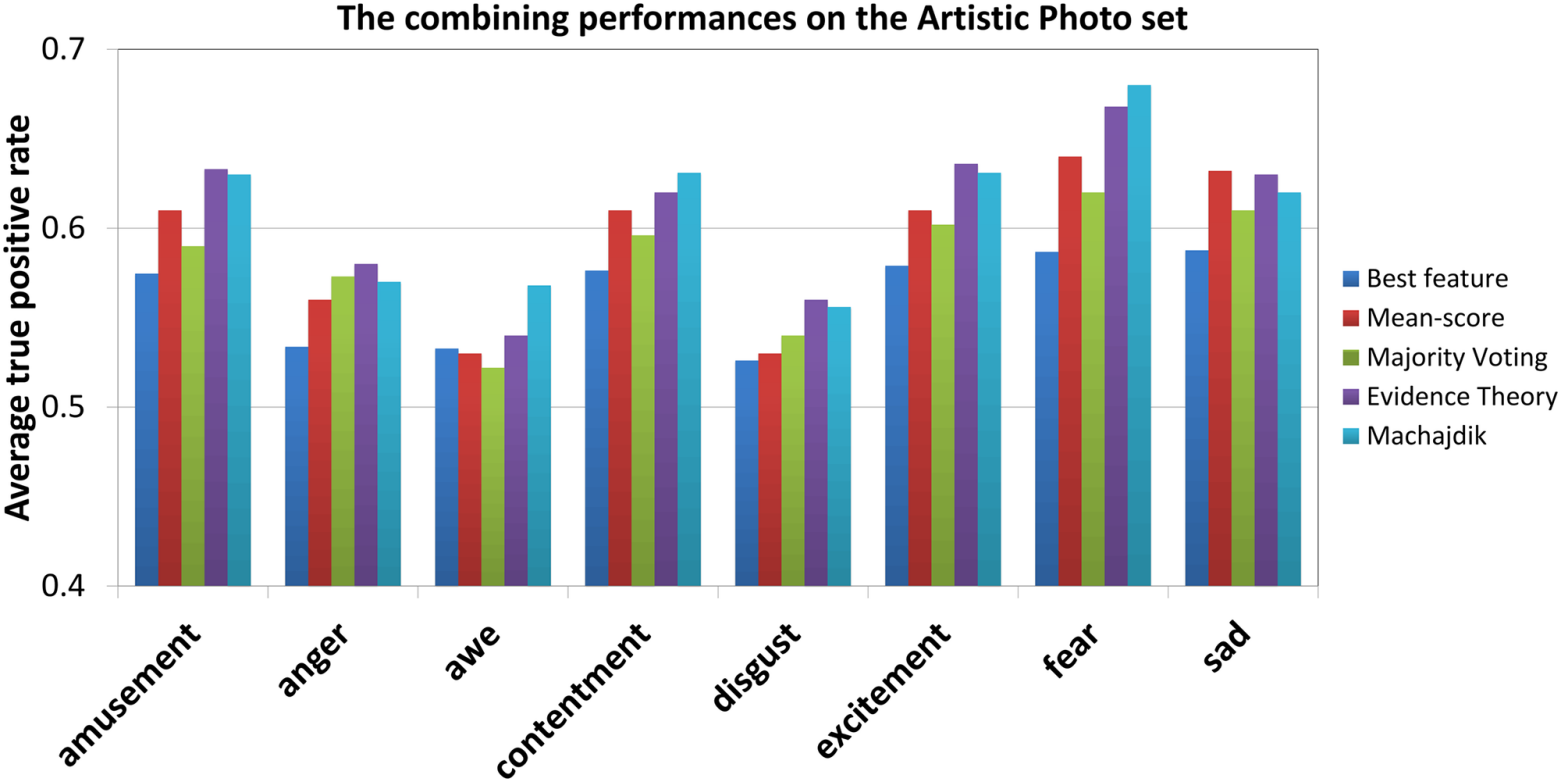
Performance of the various combination methods on the artistic photo Set.

Fig 8 shows the combining results on Artistic Photo set. It is clear that the performances of D-S evidence theory fusion method with average 63% ATPR outperforms the best individual feature and the other conventional methods. It also performs better than the state-of-art [5] in the cases of “Amusement”, “Anger”, “Disgust”, “Excitement”and “Sad”. This further demonstrate that the D-S evidence theory fusion approach has the ability to fuse different ambiguous sources of information for affective concepts, and can effectively improves the performance of VAC task.

Table 4 shows the combing results on MirFlickr Affect dataset based on D-S evidence theory. It shows that the visual features perform better than textual features with average 4% ATPR, and the eHTC outperforms the eMTC except at the neutral valence class. Specifically, the fusion of mid-level group and text features perform better on the valence dimension, while the combination of color features and text features work well on the arousal dimension. When combined with textual features, the performance of the shape feature group improves obviously with average 4% ATPR improvement. Moreover, the combination of all visual and textual features achieves the best classification accuracy for all classes. These results indicate that the proposed textual features can help to improve the performance of conventional visual based affective classification by employing the D-S evidence theory fusion approach, which exploits the complementary information provided by the different classifiers.

**Table 4:** The performance with different settings on MirFlickr affect set by combining textual and visual features based on the D-S evidence theory. The best performance in each panel is indicated in bold.

	LV(%)	NV(%)	HV(%)	LA(%)	NA(%)	HA(%)
*txtf_eMTC*	**43.3**	49.8	**54.6**	**45.2**	**53.4**	**48.8**
*txtf_eHTC*	51.4	**53.1**	56.1	48.7	56.4	52.8
Color	51.2	48.6	50.5	58.6	59.3	56.4
Color+*txtf_eMTC*	**53.5**	**48.8**	51.5	**58.3**	**59.5**	56.3
Color+*txtf_eHTC*	52.4	49.5	**52.4**	59.7	60.5	**57.5**
Texture	50.5	49.3	50.2	53.2	52.1	52.5
Texture+*txtf_eMTC*	**51.2**	**50.7**	**50.4**	**55.5**	**53.4**	**53.7**
Texture+*txtf_eHTC*	54.3	53.6	52.1	53.1	54.5	56.7
Shape	49.2	47.8	48.1	51.2	48.5	48.0
Shape+*txtf_eMTC*	**50.1**	**47.5**	**51.6**	**52.4**	**48.8**	**52.1**
Shape+*txtf_eHTC*	52.3	50.3	53.8	52.2	52.5	53.8
Mid-level	55.6	54.2	55.8	54.5	55.3	53.4
Mid-level+*txtf_eMTC*	55.3	56.2	**56.7**	**55.2**	**56.8**	**54.5**
Mid-level+*txtf_eHTC*	**57.3**	**57.6**	58.3	57.5	58.4	56.2
All visual	57.8	55.3	56.4	60.7	60.2	59.8
All visual+*txtf_eMTC*	58.2	59.3	58.6	63.5	63.3	61.4
All visual+*txtf_eHTC*	56.6	58.9	46.5	59.1	62.4	59.1
All visual+ All text	**59.9**	**65.4**	**55.6**	**64.5**	**64.5**	**63.2**

## Discussion

With the rapid development in social networks, there is a constantly focus on utilizing multimedia resources to accomplish machine-learning tasks. Indeed, not only the images can help to text-based analysis, e.g. sentiment analysis [84, 85], but also the texts can improve image-based classification, such as VOC task [86–88] These works show that the multimodal approaches can combine the preponderance and complementary information of each sources, and achieve better classification results than single modality, which is also confirmed by our results on VAC task.

The limitations of our approach involve following issues. First, the small size of training data sets makes the modalities trained insufficiency. To overcome this, we have employed 5-fold cross validation, but leaving with unstable standard deviations in the small training size classes. Second, the proposed fusion approach based on D-S evidence theory performs well for VAC task, but having complex parameters to be tuned and causing a time-consuming train process. Third, the texts from social networks usually include informal expressions, e.g Emoji, but our text features ignore them during the preprocessing stage.

## Conclusion and future work

In this paper, we present a multimodal approach for the VAC task. Firstly, we proposed two text-based features to capture emotional semantics from image tags. We also evaluated various visual features, aiming at characterizing visual content related with emotional concepts. Finally, we employed a fusion method based on the D-S theory of evidence, which exploits the complementary information to resolve the conflicts between classifiers.

The experiments were conducted on three databases: the IAPS, the Artistic Photo set and MirFlickr Affect set, and have shown promising results on visual affective classification. From the results, we can conclude as follows: (i) the fused method based on D-S evidence theory is proved to be useful for the VAC task in efficiently fusing different features; (ii) the proposed textual eHTC can effectively capture emotional semantics from image tags, and help to improve the performance of visual classifiers for VAC task; (iii) the classification of visual emotional concepts is still extremely challenging, and the average performance of this approach is 57%, which can be expected as the small training set.

In the future, we plan to make more efforts on the following aspects: building a large database, exploring regression model for VAC in a dimensional emotion model, and testifying Word2Vector or other similarities to improve eHTC. At last, how to efficiently apply the proposed methods to web-images on a large scale will also be investigated.

## References

[1] Picard RW. Affective computing. MIT press; 2000.

[2] Zeng Z, Pantic M, Roisman GI, Huang TS. A Survey of Affect Recognition Methods: Audio, Visual, and Spontaneous Expressions. IEEE Trans Pattern Anal Mach Intell. 2009; p. 39–58. 10.1109/TPAMI.2008.52 19029545

[3] Wang W, He Q. A survey on emotional semantic image retrieval. In: ICIP; 2008. p. 117–120.

[4] Bradley MM, Lang PJ. Measuring emotion: The self-assessment manikin and the semantic differential. Journal of Behavior Therapy and Experimental Psychiatry. 1994;25(1):49–59. 10.1016/0005-7916(94)90063-9 7962581

[5] Machajdik J, Hanbury A. Affective image classification using features inspired by psychology and art theory. In: Proceedings of the international conference on Multimedia. ACM; 2010. p. 83–92.

[6] Alameda-Pineda X, Ricci E, Yan Y, Sebe N. Recognizing Emotions from Abstract Paintings using Non-Linear Matrix Completion. In: IEEE International Conference on Computer Vision and Pattern Recognition. Las Vegas, USA; 2016.

[7] Peng KC, Chen T, Sadovnik A, Gallagher AC. A Mixed Bag of Emotions: Model, Predict, and Transfer Emotion Distributions. In: The IEEE Conference on Computer Vision and Pattern Recognition (CVPR); 2015.

[8] Liu N, Dellandréa E, Tellez B, Chen L. Evaluation of Features and Combination Approaches for the Classification of Emotional Semantics in Images. In: International Conference on Computer Vision, Theory and Applications (VISAPP); 2011.

[9] Liu N, Dellandréa E, Tellez B, Chen L, Chen L. Associating Textual Features with Visual Ones to Improve Affective Image Classification. In: ACII (1); 2011. p. 195–204.

[10] Valdez P, Mehrabian A. Effects of color on emotions. J Exp Psychol Gen. 1994;123:394–409. 10.1037/0096-3445.123.4.394 7996122

[11] Colombo C, Bimbo AD, Pala P. Semantics in Visual Information Retrieval. IEEE Multimedia. 1999;6:38–53. 10.1109/93.790610

[12] Tamura H, Mori S, Yamawaki T. Texture features corresponding to visual perception. IEEE Transactions on System, Man and Cybernatic. 1978;6.

[13] Datta R, Li J, Wang JZ. Content-based image retrieval: approaches and trends of the new age. In: Multimedia Information Retrieval; 2005. p. 253–262.

[14] Ke Y, Tang X, Jing F. The design of high-level features for photo quality assessment. In: Computer Vision and Pattern Recognition, 2006 IEEE Computer Society Conference on. vol. 1. IEEE; 2006. p. 419–426.

[15] Itten J, Van Haagen E. The art of color: the subjective experience and objective rationale of color. Van Nostrand Reinhold New York, NY, USA; 1973.

[16] Liu S, Xu D, Feng S. Emotion categorization using affective-pLSA model. Optical Engineering. 2010;49(12):127201–127201–12 10.1117/1.3518051

[17] Wang G, Hoiem D, Forsyth DA. Building text features for object image classification. In: CVPR; 2009. p. 1367–1374.

[18] Sivic J, Zisserman A. Video Google: A Text Retrieval Approach to Object Matching in Videos. In: ICCV; 2003. p. 1470–1477.

[19] Guillaumin M, Verbeek JJ, Schmid C. Multimodal semi-supervised learning for image classification. In: CVPR; 2010. p. 902–909.

[20] Liu N, Zhang Y, Dellandréa E, Bres S, Chen L. LIRIS-Imagine at ImageCLEF 2012 Photo Annotation task. In: CLEF Workshop Notebook Paper; 2012.

[21] Liu N, Dellandra E, Chen L, Zhu C, Zhang Y, Bichot CE, et al Multimodal Recognition of Visual Concepts using Histograms of Textual Concepts and Selective Weighted Late Fusion Scheme. Computer Vision and Image Understanding. 2013;117(5):493–512. 10.1016/j.cviu.2012.10.009

[22] Lang PJ, Bradley MM, Cuthbert BN. International affective picture system (IAPS): Technical manual and affective ratings; 1999.

[23] Hevner K. Experimental studies of the elements of expression in music. The American Journal of Psychology. 1936;48(2):246–268. 10.2307/1415746

[24] Osgood CE, Suci G, Tannenbaum PH. et al The measurement of meaning. 1957; p. 189–216.

[25] Dunker P, Nowak S, Begau A, Lanz C. Content-based mood classification for photos and music: a generic multi-modal classification framework and evaluation approach. In: Multimedia Information Retrieval; 2008. p. 97–104.

[26] Feist A, Stephan E. Entwicklung eines Verfahrens zur Erfassung des Gefühlszustandes (VGZ); 2007.

[27] Li B, Xiong W, Hu W, Ding X. Context-aware affective images classification based on bilayer sparse representation. In: Proceedings of the 20th ACM international conference on Multimedia. MM’12; 2012. p. 721–724.

[28] Columbo C, Pala P, Del Bimbo A. Semantics in visual information retrieval. IEEE Multimedia. 1999;6(3):38–53. 10.1109/93.790610

[29] Wang Wei-ning JSm Yu Ying-lin. Image Retrieval by Emotional Semantics: A Study of Emotional Space and Feature Extraction. In: International Conference on Systems, Man and Cybernetics. vol. 4; 2006. p. 3534–3539.

[30] Hinton GE, Salakhutdinov RR. Reducing the Dimensionality of Data with Neural Networks. Science. 2006;313(5786):504 10.1126/science.1127647 16873662

[31] Karpathy A, Toderici G, Shetty S, Leung T, Sukthankar R, Li FF. Large-Scale Video Classification with Convolutional Neural Networks. In: IEEE Conference on Computer Vision and Pattern Recognition; 2014. p. 1725–1732.

[32] Li FF, Bradski G, Yao B. A codebook-free and annotation-free approach for fine-grained image categorization. In: IEEE Conference on Computer Vision and Pattern Recognition; 2012. p. 3466–3473.

[33] Jia D, Krause J, Li FF. Fine-Grained Crowdsourcing for Fine-Grained Recognition. 2013;9(4):580–587.

[34] Hinton GE, Osindero S. A fast learning algorithm for deep belief nets. In: Neural Computation; 2006 p. 2006. 10.1162/neco.2006.18.7.1527 16764513

[35] Yangqing Jia, Evan Shelhamer, Jeff Donahue, et al Caffe: Convolutional Architecture for Fast Feature Embedding. Eprint Arxiv. 2014; p. 675–678.

[36] Krizhevsky A, Sutskever I, Hinton GE. ImageNet classification with deep convolutional neural networks. In: International Conference on Neural Information Processing Systems; 2012. p. 1097–1105.

[37] Lecun Y HG Bengio Y. Deep learning. Nature. 2015; p. 436–444. 10.1038/nature14539 26017442

[38] Deng J, Dong W, Socher R, Li LJ, Li K, Li FF. ImageNet: A large-scale hierarchical image database. In: Computer Vision and Pattern Recognition, 2009. CVPR 2009. IEEE Conference on; 2009. p. 248–255.

[39] Awad FJMMJDKWSAFOR G. TRECVID 2016: Evaluating Video Search, Video Event Detection, Localization, and Hyperlinking. In: Proceedings of TRECVID; 2016.

[40] Bänziger T, Grandjean D, Scherer KR. Emotion recognition from expressions in face, voice, and body: the Multimodal Emotion Recognition Test (MERT). Emotion. 2009;9(9):691–704. 19803591 10.1037/a0017088

[41] Kim Y, Lee H, Provost EM. Deep learning for robust feature generation in audiovisual emotion recognition. In: 2013 IEEE International Conference on Acoustics, Speech and Signal Processing; 2013. p. 3687–3691.

[42] Ngiam J, Khosla A, Kim M, Nam J, Lee H, Ng AY. Multimodal Deep Learning. In: International Conference on Machine Learning, ICML 2011, Bellevue, Washington, Usa, June 28–July; 2011. p. 689–696.

[43] Liu W, Zheng W, Lu B. Multimodal Emotion Recognition Using Multimodal Deep Learning. CoRR. 2016.

[44] Ballester P, Araujo RM. On the Performance of GoogLeNet and AlexNet Applied to Sketches;.

[45] Malinowski M, Fritz M. A Multi-World Approach to Question Answering about Real-World Scenes based on Uncertain Input. In: Advances in neural information processing systems; 2014.

[46] Sohn K, Shang W, Lee H. Improved Multimodal Deep Learning with Variation of Information. In: Advances in Neural Information Processing Systems; 2014 p. 2141–2149.

[47] Mensink T, Csurka G, Perronnin F, Sáanchez J, Verbeek JJ. LEAR and XRCE’s Participation to Visual Concept Detection Task—ImageCLEF 2010. In: CLEF Workshop Notebook Paper; 2010.

[48] Binder A, Samek W, Kloft M, Müller C, Müller KR, Kawanabe M. The Joint Submission of the TU Berlin and Fraunhofer FIRST (TUBFI) to the ImageCLEF2011 Photo Annotation Task. In: CLEF Workshop Notebook Paper; 2011.

[49] Dumais ST. Latent Semantic Analysis; 2005.

[50] Hofmann T. Probabilistic latent semantic indexing. In: Proceedings of the 22nd annual international ACM SIGIR conference on Research and development in information retrieval; 1999. p. 50–57.

[51] Blei DM, Ng AY, Jordan MI. Latent dirichlet allocation. J Mach Learn Res. 2003;3:993–1022.

[52] Mikolov T, Chen K, Corrado G, Dean J. Efficient Estimation of Word Representations in Vector Space. Computer Science. 2013;.

[53] Jurgens DA, Turney PD, Mohammad SM, Holyoak KJ. SemEval-2012 task 2: measuring degrees of relational similarity. In: Joint Conference on Lexical and Computational Semantics; 2012. p. 356–364.

[54] Zhila A. Combining Heterogeneous Models for Measuring Relational Similarity; 2013 p. 1000–1009.

[55] Maas AL, Daly RE, Pham PT, Huang D, Ng AY, Potts C. Learning word vectors for sentiment analysis. In: The Meeting of the Association for Computational Linguistics: Human Language Technologies, Proceedings of the Conference; 2011. p. 142–150.

[56] V Yanulevskaya ea J C Van Gemert. Emotional valence categorization using holistic image features. ICIP. 2008; p. 101–104.

[57] Guo Y, Gao H. Emotion Recognition System in Images Based On Fuzzy Neural Network and HMM. In: IEEE ICCI. IEEE; 2006. p. 73–78.

[58] Malinowski M, Fritz M. Hard to Cheat: A Turing Test based on Answering Questions about Images. Computer Science. 2015;.

[59] Malinowski M, Rohrbach M, Fritz M. Ask Your Neurons: A Neural-Based Approach to Answering Questions about Images. 2015; p. 1–9.

[60] Scherer K. Appraisal Processes in Emotion: Theory, Methods, Research (Series in Affective Science). Oxford University Press, USA; 2001.

[61] Bradley MM, Lang PJ. Affective norms for English words (ANEW): Stimuli, instruction manual, and affective ratings. Gainesville, Florida: Center for Research in Psychophysiology, University of Florida; 1999.

[62] McNair D M LM Droppleman L F. Edits manual for the profile of mood states: POMS. 1992;.

[63] Thelwall M, Buckley K, Paltoglou G, Cai D, Kappas A. Sentiment strength detection in short informal text. Journal of the American Society for Information Science and Technology. 2010;61(12):2544–2558. 10.1002/asi.21416

[64] Esuli A, Sebastiani F. SentiWordNet: A Publicly Available Lexical Resource for Opinion Mining. In: In In Proceedings of the 5th Conference on Language Resources and Evaluation; 2006. p. 417–422.

[65] Resnik P. Using Information Content to Evaluate Semantic Similarity in a Taxonomy. CoRR. 1995.

[66] Steven Bird EL Ewan Klein. Natural Language Processing with Python. O’Reilly Media; 2009.

[67] Sussna M. Word Sense Disambiguation for Free-text Indexing Using a Massive Semantic Network. In: Proceedings of the Second International Conference on Information and Knowledge Management; 1993. p. 67–74.

[68] Wu Z, Palmer M. Verb Semantics and Lexical Selection. CoRR. 1994.

[69] Lin D. An information-theoretic definition of similarity. In: ICML; 1998 p. 296–304.

[70] Solli M, Lenz R. Emotion related structures in large image databases. In: CIVR; 2010. p. 398–405.

[71] Haralick RM. Statistical and Structural Approaches to Texture. Proceedings of the IEEE. 1979;67:786–804. 10.1109/PROC.1979.11328

[72] Ojala T, Pietikäinen M, Harwood D. A comparative study of texture measures with classification based on featured distributions. Pattern Recognition. 1996; p. 51–59. 10.1016/0031-3203(95)00067-4

[73] Pujol A, Chen L. Line segment based edge feature using Hough transform. In: The Seventh IASTED International Conference on Visualization, Imaging and Image Processing. VIIP’07; 2007. p. 201–206.

[74] Datta R, Joshi D, Li J, Wang JZ. Studying aesthetics in photographic images using a computational approach. In: Proceedings of the 9th European conference on Computer Vision. vol. 3 of ECCV; 2006. p. 288–301.

[75] Viola PA, Jones MJ. Robust Real-Time Face Detection. In: ICCV. vol. 57; 2001. p. 137–154.

[76] Wang Y, Ai H, Wu B, Huang C. Real time facial expression recognition with AdaBoost. In: Proceedings of the 17th ICPR. vol. 3; 2004. p. 926–929 Vol.3.

[77] Yanulevskaya V, Van Gemert JC, Roth K, Herbold AK, Sebe N, Geusebroek JM. Emotional valence categorization using holistic image features. In: Image Processing, 2008. ICIP 2008. 15th IEEE International Conference on; 2008. p. 101–104.

[78] deviantart;. www.deviantart.com.

[79] Dellandréa E, Liu N, Chen L. Classification of affective semantics in images based on discrete and dimensional models of emotions. In: International Workshop on Content-Based Multimedia Indexing (CBMI); 2010. p. 99–104.

[80] Huiskes MJ, Lew MS, Lew MS. The MIR flickr retrieval evaluation In: Multimedia Information Retrieval; 2008 p. 39–43.

[81] Mikels J, Fredrickson B, Larkin G, Lindberg C, Maglio S, Reuter-Lorenz P. Emotional category data on images from the international affective picture system. Behavior Research Methods. 2005;37(4):626–630. 10.3758/BF03192732 16629294 PMC1808555

[82] Chang CC, Lin CJ. LIBSVM: A library for support vector machines. ACM Transactions on Intelligent Systems and Technology. 2011;2:1–27.

[83] Al-Ani A, Deriche M. A New Technique for Combining Multiple Classifiers Using the Dempster-Shafer Theory of Evidence. Journal of Artificial Intelligence Research. 2002;17:333–361.

[84] Poria S, Cambria E, Howard N, Huang GB, Hussain A. Fusing audio, visual and textual clues for sentiment analysis from multimodal content. Neurocomputing. 2016;174:50–59. 10.1016/j.neucom.2015.01.095

[85] You Q, Cao L, Cong Y, Zhang X. A Multifaceted Approach to Social Multimedia-Based Prediction of Elections. IEEE Transactions on Multimedia. 2015;17(12):2271–2280. 10.1109/TMM.2015.2487863

[86] Wang G, Forsyth D. Object image retrieval by exploiting online knowledge resources. In: Computer Vision and Pattern Recognition, 2008. CVPR 2008. IEEE Conference on; 2008. p. 1–8.

[87] Wang Y, Chen J, Ningning Liu by title = BUAA-iCC at ImageCLEF 2015 Scalable Concept Image Annotation Challenge;.

[88] Liu N, Zhang Y, Dellandréa E, Bres S, Chen L. LIRIS-Imagine at ImageCLEF 2011 Photo Annotation task. In: CLEF Workshop Notebook Paper; 2011.

